# Synaptic vs. Non-Synaptic Glycine Receptors: Physiological Role and Implications in Alzheimer’s Disease Pathology

**DOI:** 10.3390/ijms27146306

**Published:** 2026-07-15

**Authors:** Eva Kiss, Joachim Kirsch, Stefan Kins, Jochen Kuhse

**Affiliations:** 1 Department of Genetics, Cell and Molecular Biology, George Emil Palade University of Medicine, Pharmacy, Science and Technology of Târgu Mures, 540142 Târgu Mures, Romania; 2 Institute of Anatomy and Cell Biology, University of Heidelberg, 69120 Heidelberg, Germany; joachim.kirsch@uni-heidelberg.de (J.K.); jochen.kuhse@t-online.de (J.K.); 3 Department of Human Biology and Human Genetics, University of Kaiserslautern, 67663 Kaiserslautern, Germany; kins@rptu.de

**Keywords:** extrasynaptic GlyRs, phasic vs. tonic inhibition, Alzheimer’s disease, neuroprotection

## Abstract

Strychnine-sensitive glycine receptors (GlyRs) are pentameric ligand-gated chloride channels that mediate fast inhibitory neurotransmission in the central nervous system (CNS), with high expression in the spinal cord, brainstem, cerebellum, and retina. Beyond traditional postsynaptic phasic inhibition, emerging evidence highlights the importance of extrasynaptic GlyRs—expressed in both neuronal and non-neuronal cells—in mediating tonic inhibition by sensing ambient glycine levels, including in the forebrain. These non-synaptic receptors display high agonist affinity, unique subunit compositions, and distinct pharmacodynamics. Notably, recent studies have begun to implicate aberrant GlyR signaling in Alzheimer’s disease (AD) pathology; however, its functional role in this specific neurodegenerative context remains only poorly understood. This review synthesizes the molecular properties and functional significance of these diverse GlyR populations, emphasizing their involvement in calcium signaling, inhibitory tone, and neural circuit modulation, while critically evaluating their emerging therapeutic potential in AD.

## 1. Introduction

**Figure 1:**
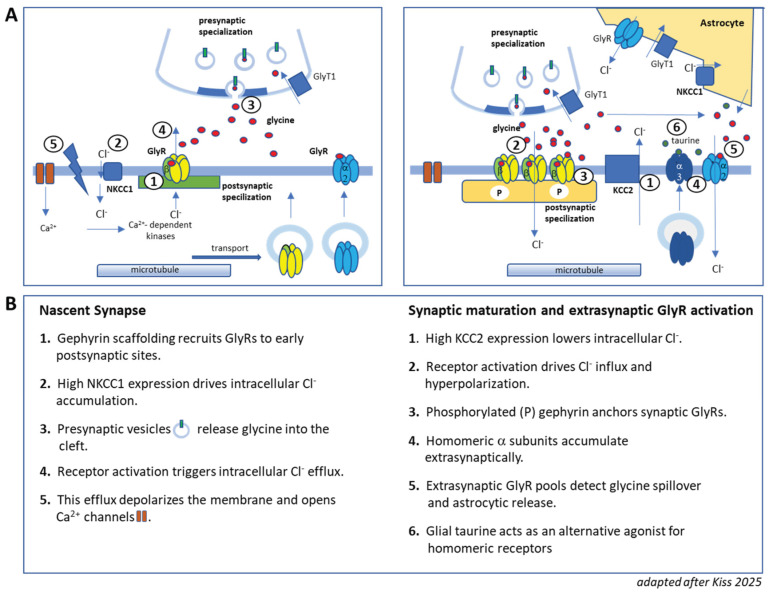
**Different stages of glycinergic synapse formation and activation of extrasynaptic GlyRs.** Synapses initiate as nascent contacts that mature into plastic synaptic connections. (**A**) Schematic view of a nascent glycinergic synapse (**left**) and a mature glycinergic synapse (**right**). In nascent synapses, ion channel activation causes an outward Cl^−^ current due to high intracellular Cl^−^ concentrations driven by NKCC1 activity. This depolarization opens voltage-dependent Ca^2+^ channels, activating Ca^2+^-dependent kinases required for early synaptic remodeling. In mature synapses, GlyR activation drives a hyperpolarizing inward chloride current due to low intracellular Cl^−^ levels maintained by KCC2 activity. Synaptic maturation depends on active intracellular trafficking; both synaptic heteromeric and homomeric extrasynaptic GlyRs are packaged into transport vesicles and transported along the microtubule network for plasma membrane insertion. Once at the membrane, an enlarged gephyrin scaffold mechanically anchors a high density of synaptic heteromeric GlyRs harboring the structural β subunit, which directly mediates gephyrin binding. The stability of this postsynaptic domain is dynamically regulated by gephyrin phosphorylation (P), which dictates scaffold clustering and controls receptor retention or lateral diffusion. Concurrently, distinct extrasynaptic assemblies of homomeric α2 and α3 GlyRs are structurally segregated outside the postsynaptic density. These mobile receptors are activated by glycine spillover during intense firing or ambient glial secretion. Additionally, these extrasynaptic populations may be modulated by taurine released from astrocytes via specific volume-regulated membrane transport proteins to fine-tune baseline network excitability. (**B**) Short description of the events presented in (**A**). Further mechanistic details are provided in the manuscript text. NKCC1: Natrium-Kalium-Chlorid-Cotransporter 1; KCC2: Kalium-Chlorid-Cotransporter 2.

The strychnine-sensitive glycine receptors (GlyRs) serve as fundamental mediators of inhibitory control throughout the central nervous system. While traditionally defined as postsynaptic anchors for fast inhibition, GlyRs exhibit diverse functional profiles depending on their specific subcellular localization and signaling mode across neuronal and glial populations. Postsynaptic heteromeric αβ complexes require gephyrin-mediated anchoring to drive Cl^−^ influx in mature neurons, resulting in membrane hyperpolarization and reduced excitability [1] (Figure 1). Conversely, a substantial pool of GlyRs is localized at extrasynaptic sites on the dendritic shaft, soma or axon, where they contribute to persistent, tonic inhibitory conductance [2] (Figure 1). Activated by glycine diffusing from the synapse or by baseline extracellular fluid levels, this tonic inhibition selectively decreases the amplitude and duration of excitatory postsynaptic potentials, thereby reducing the probability that an action potential will be generated [3,4].

These distinct compartmental roles correlate with specific structural configurations, as presynaptic and extrasynaptic signaling are more frequently associated with homomeric α-assemblies (Figure 1). Because three functional human α subunits (α1, α2, α3) exist, each expressed across multiple splice variants, the precise pentameric assembly of these distinct subunits drives extensive functional heterogeneity among GlyR populations [5]. Pharmacologically, GlyRs are defined by their high sensitivity to strychnine and their relative insensitivity to the gamma-aminobutyric acid type A receptor (GABA_A_R) antagonist bicuculline, allowing for their functional isolation within complex neural networks. As one of the primary regulators of network excitability and information processing, GlyRs—much like GABA_A_Rs—are key players in both physiological and pathological contexts. Beyond their classical roles in motor coordination, muscle tone, and respiratory rhythm [1,6], specific subunits mediate highly specialized biological processes throughout the CNS. For instance, α2-containing GlyRs are essential for neurogenesis and proper cortical migration during development [7,8]. In contrast, α3 subunits are primary mediators of breathing control and hearing function [9,10,11], while also playing a central role in the modulation of pain signaling [6,12,13]. Given these critical functions, the dysregulation of these specific isoforms has been identified as a hallmark of several neurological conditions, including autism [14,15], epilepsy [16,17], and alcohol addiction [18,19,20]. Specifically, a comprehensive review by Fraser and Harvey [21] summarized recent findings, highlighting that GLRA2—the gene encoding the α2 subunit—microdeletions and missense variants are often accompanied by epilepsy, language delay, and autism spectrum disorder. While these roles seem to be well-documented in sensory and motor circuits, the contribution of the glycinergic system to higher-order cognitive stability is increasingly recognized. This becomes particularly relevant in neurodegenerative diseases such as Alzheimer’s disease (AD), where the delicate balance between excitation and inhibition is profoundly disrupted [22,23]. Such disruption seems not only to manifest as a significantly increased risk of subclinical epilepsy in AD patients but also to establish a bidirectional relationship, as individuals with epilepsy face a substantially higher risk of developing dementia later in life [24,25], a relationship formally assessed in a recent meta-analysis by Chen et al. [26].

This review aims to synthesize how the molecular structure and subcellular localization of GlyRs dictate their physiological roles, and how their breakdown might contribute to the network instability and cognitive decline characteristic of AD. Ultimately, this work proposes the restoration of glycinergic inhibition as a significant, yet under-explored, therapeutic target in the treatment of AD.

## 2. Structural Biology and Regulatory Mechanisms of GlyR Pentamers

### 2.1. Molecular Characterization and Composition of GlyRs

The identification of the GlyR as the first neurotransmitter receptor purified from the mammalian central nervous system marked a milestone in neurobiology, achieved through affinity chromatography [27]. This process revealed a structural complex composed of α and β subunits—with a molecular mass of 48/49 kDa and 58 kDa, respectively—anchored by the 93 kDa scaffolding protein gephyrin at the postsynaptic membrane specializations [28,29]. Subsequent molecular cloning and biochemical analyses identified three functional α subunits in rats and humans (α1, α2, and α3) [28,30,31], while the α4 subtype was characterized as a pseudogene in humans despite remaining active in other species [32,33].

The GlyRs are members of the Cys-loop family of pentameric ligand-gated ion channels, which also includes gamma-aminobutyric acid type A (GABA_A_), nicotinic acetylcholine (nACh), and 5-hydroxytryptamine type-3 (5-HT3) receptors [34]. The term “Cys-loop” refers to a signature disulfide bond structure located within the N-terminal extracellular domain [35]. Each receptor subunit is characterized by a large N-terminal extracellular domain, which harbors the ligand-binding sites and translates agonist binding into the conformational changes required for channel opening. This is followed by four transmembrane domains (TM1–TM4), with a large intracellular loop between TM3 and TM4 that terminates in a smaller C-terminal extracellular region. The ion-conducting pore is specifically lined by the TM2 domains, which determine the receptor’s selectivity for Cl^−^ and dictate the sensitivity of homomeric α GlyRs to blockers such as picrotoxin [36,37].

The structural assembly of GlyRs varies between homomeric and heteromeric configurations (Figure 1). Although the primary sequences of α and β subunits allow for different complexes, traditional biochemical models originally favored a fixed stoichiometry of three α subunits to two β subunits [38]. However, subsequent structural and biochemical analysis, culminating in high-resolution cryo-EM studies, established that native he-teromeric receptors predominantly adopt a fixed architecture of four α subunits to one β subunit (4α:1β) [39]. The exact number and specific type of subunits embedded within these pentameric assemblies directly dictate the receptor’s overall biophysical properties. Because the β subunit itself does not form an efficiently conducting pore, the single-channel conductance is strictly determined by which α variants line the channel lumen. Notably, receptors incorporating α2 or α3 subunits exhibit a larger single-channel conductance (approx. 111 pS and 105 pS, respectively) compared to the more common α1-containing complexes (approx. 86 pS), facilitating highly specialized inhibitory signaling [40]. When heteromerized with the β subunit, these conductances drop substantially to a range between 44 and 54 pS, demonstrating a dominant-negative biophysical influence of the β subunit on ion permeation. Structural insights have elucidated the molecular basis for this conductance decrease, revealing that the β subunit introduces specific residues into the channel pore. These include negatively charged glutamate 290 and glutamate 297, alongside the bulky phenylalanine 282 side chain, which create electrostatic and physical barriers that restrict Cl^−^ flow [37].

### 2.2. Post-Transcriptional Diversity: Alternative Splicing and Editing of GlyR Subunits

Beyond subunit assembly, the functional profile of GlyRs is significantly expanded by alternative splicing and mRNA editing, allowing a single gene to produce multiple protein variants with distinct kinetic properties and expression patterns [41,42]. For the α1 subunit, the α1-ins splice variant contains an additional eight amino acids within its large cytoplasmic loop [43]. The α2 subunit exists as α2A and α2B variants, which were identified by homology cloning and reveal amino acid variations in the extracellular domain [31]. Similarly, the α3 subunit produces α3K and α3L variants, distinguished by a 15-amino acid insert (TEAFALEKFYRFSDT) in the cytoplasmic loop that causes differences in receptor motion and desensitization kinetics [44]. These receptor variants exhibit critical functional differences that dictate their specific roles in the brain (Figure 1). For instance, the embryonic form of the GlyR—a homomer composed of five α2 subunits—is expressed before the formation of mature synapses. Its properties are specifically adapted for non-synaptic signaling, where glycine is released in a diffuse, paracrine manner rather than in sharp, localized pulses [45]. A key distinction lies in the receptor’s activation mechanics: because these homomeric receptors have a relatively low opening probability, they are unlikely to be effectively activated by the rapid neurotransmitter release at mature synapses. Instead, they feature an exceptionally high potency for glycine, allowing them to detect and respond to the low, ambient levels of agonists present in the embryonic environment. This specialization is most evident in the α2B splice variant. Through amino acid substitutions at positions 58 and 59 (Val58Ile and Thr59Ala relative to α2A), the α2B variant achieves a significantly higher potency for glycine compared to the α2A form, displaying an EC50 of 34 μM compared to 66 μM for α2A, and roughly 190 μM for mature synaptic α1β heteromeric GlyRs. Furthermore, this structural change increases the receptor’s sensitivity to partial agonists such as β-alanine and taurine, which are prevalent during early development [46]. Collectively, these features ensure that α2-containing receptors maintain essential signaling during the structural assembly of the nervous system, long before the establishment of classical synaptic transmission.

The genetically encoded information of GlyRs can be further modified through RNA editing, primarily via the enzymatic deamination of adenosine and cytidine. These processes are mediated by adenosine deaminases acting on RNA (ADAR) and apolipoprotein B mRNA editing complexes (APOBEC), respectively [47]. The resulting substitutions of inosine or uracil can lead to amino acid changes that diversify the proteome or even “correct” the genomic code.

In GlyRs, specific amino acid substitutions in the orthosteric binding site within the extracellular domain have been shown to significantly alter agonist affinity. Specifically, a proline-to-leucine substitution resulting from C-to-U editing of α3 transcripts in Xenopus oocytes and primary hippocampal neurons increased the apparent affinity for both glycine and taurine without modifying desensitization kinetics. This editing process was highly dynamic, as evidenced by the pilocarpine-induced lesion model, where editing levels rose as a homeostatic response to stabilize hyper-excitable hippocampal networks [42]. Similar editing of α2 transcripts (GLRA2) at the corresponding position (192) also produced receptors with increased agonist affinity [16]. Together, the resulting splice variants and edited transcripts result in high-affinity GlyRs ideally suited to function as extrasynaptic ion channels.

### 2.3. Structural States and Gating of GlyRs

GlyRs undergo transitions between distinct structural states to control Cl^−^ influx, a process profoundly elucidated by X-ray crystallography and high-resolution cryo-electron microscopy as reviewed recently by Zhu [48]. Early studies on the human GlyR α3 isoform provided the inaugural molecular models of transitions between resting and active conformations [49,50]. This characterization was subsequently expanded by cryo-electron microscopy of zebrafish α1, resolving the receptor in various functional states: strychnine-bound (closed), glycine-bound (open), and glycine–ivermectin-bound (modulated/desensitized) [51,52]. The orthosteric binding site is situated at the subunit interface, where ligand recognition is mediated by highly conserved, oppositely charged residues. Within this pocket, aromatic residues provide essential stabilization; specifically, the amino group of glycine forms a hydrogen bond with the phenylalanine-159 carbonyl and a cation-π interaction with phenylalanine-207. Simultaneously, the carboxylate group is anchored by a hydrogen-bond network involving arginine-65, serine-129, and threonine-204. High-resolution data further revealed that this connection is strengthened by a water-mediated bridge involving glutamate-157 and serine-158, ensuring the high specificity required to trigger channel opening [37]. This intricate molecular arrangement ensures the exquisite selectivity required to distinguish glycine from similar metabolites, while providing the mechanical energy necessary to trigger the conformational shift that opens the ion pore.

Within the human α1 homomeric GlyR configuration, glycine serves as the endogenous full agonist, characterized by a high open probability of 97% and a potent EC50 of 190 μM stabilizing the open-channel conformation. In contrast, taurine and GABA act as cross-reactive partial agonists on this channel, exhibiting significantly lower efficacy and potency. On these assemblies, they display open probabilities of approximately 66% and 39%, and EC50 values of 1.05 mM and 28.4 mM, respectively. This variation in efficacy correlates directly with the degree of contraction within the agonist-binding pocket during transitions from agonist-bound closed intermediate to open conformations [53]. Altering these dynamics can shift selectivity; for example, a phenylalanine-to-tyrosine mutation at position 175 confers GABA sensitivity by creating a novel hydrogen bond between the agonist carboxylate group and the hydroxyl oxygen of the tyrosine residue [53,54].

Beyond the primary binding site, GlyRs are subject to allosteric modulation. The transition between resting (closed), open, and desensitized states is driven by a global counterclockwise rotation of the transmembrane subdomains around the pore axis [55]. This rotational wave translates ligand binding into the mechanical dilation of the transmembrane helical bundle, physically opening the gate to permit Cl^−^ permeation. Consequently, any alterations in membrane composition or elevated oxidative stress—common hallmarks of the neurodegenerative brain—may directly disrupt the biophysical integrity of this rotational wave and the precise contraction of the binding pocket, thereby impairing overall receptor functionality and inhibitory neurotransmission.

### 2.4. Dynamics of GlyRs

Lateral diffusion drives the continuous exchange of GlyRs between synaptic and extrasynaptic compartments [56], as receptors move within the plasma membrane via Brownian motion, a process first visualized through single-quantum dot tracking [57]. This movement follows a diffusion-trap mechanism, where receptors diffusing in the extrasynaptic space are transiently captured at the postsynaptic density through direct interactions between the gephyrin E-domain and the GlyR β-subunit [58] (Figure 1). The specific mechanisms that target receptors from intracellular or extrasynaptic compartments and keep them extrasynaptic or at synapses remain incompletely understood; however, in particular, the synaptic localization of GlyRs is thought to depend on signal-dependent activation of the receptor [59].

The kinetics of this exchange—transitioning from a free-diffusing to a confined state within seconds—allow for significantly faster synaptic remodeling than traditional vesicular trafficking, yet the steady-state concentration of GlyRs is further regulated by the dynamic balance of receptor constitutive endocytosis and internalization, primarily targeting the mobile extrasynaptic receptor pool [60]. Recent research has significantly expanded the classical model established by Charrier et al. [61] regarding how the cytoskeleton regulates receptor mobility. While microtubules facilitate long-range transport and actin filaments provide local confinement, this mobility is a highly regulated process driven by biochemical switches and molecular competition. As demonstrated by Zhou et al. [62], the phosphorylation of the scaffolding protein gephyrin at serine-270 triggers its detachment from the microtubule network, consequently leading to high-affinity binding of GlyRs to synaptic gephyrin scaffolds. This reveals that microtubules act as a regulated supply line where the unloading of receptors at the synaptic periphery is actively controlled by intracellular kinases such as CDK5 and GSK-3β (glycogen synthase kinase 3 beta) [63,64].

Once receptors reach the synapse, their stay duration is governed by a competitive anchoring system. Using single-molecule tracking and magnetic tweezers, Kostrz et al. [65] established the binding energy of the isolated GlyR β subunit intracellular loop–gephyrin interaction at −13.1 kBT. This α-independent biophysical evidence suggests that GlyRs physically compete with GABA_A_Rs for a finite number of binding sites on the actin-anchored gephyrin lattice. In a crowded nano-environment, high GABA_A_R density can displace GlyRs into the faster-moving extrasynaptic pool. Finally, tracking data from Lemmens et al. [5] confirmed that α3 GlyRs are more mobile than the α1 variant. Within the actin-rich synapse, receptors move at a median rate of 0.001 μm^2^/second, but α3 subunits escape these constraints more frequently, entering the extrasynaptic membrane where diffusion speeds increase a hundredfold to 0.1 μm^2^/second.

Furthermore, lateral diffusion is highly sensitive to activity-dependent plasticity. High levels of excitatory input—particularly events triggering calcium influx via NMDA (N-methyl-D-aspartate) receptors—can decrease lateral mobility to effectively trap more GlyRs at the synapse, thereby increasing inhibitory tone [66]. Conversely, post-translational modifications, such as PKC-mediated phosphorylation at residue serine-403 of the β-subunit, can reduce gephyrin affinity, accelerating receptor escape and promoting synaptic weakening [67]. Modulating the affinity between receptors and the gephyrin scaffold provides a major mechanism for rapid homeostatic adjustment by facilitating the continuous exchange of receptors between compartments to scale inhibitory output [59].

## 3. Methodological Validation of Forebrain Extrasynaptic GlyRs

### 3.1. Anatomical Mapping and Subunit Specificity

Western blot analyses identifying characteristic bands at 48 kDa (α subunits) and 58 kDa (β subunit) [68,69], combined with immunohistochemical mapping [69,70,71], confirmed that GlyRs are distributed across the rodent and human forebrain, primarily in the striatum, hippocampus, and prefrontal cortex, where they partition into both synaptic and extrasynaptic compartments. While spinal GlyRs are primarily synaptic heteromers mediating fast phasic inhibition, the adult forebrain exhibits a high relative abundance of α2 and α3. Quantitative analyses showed that this distribution favors areas outside the synapse; roughly two-thirds of all α2- and α3-containing GlyRs in the adult hippocampus are localized to the extrasynaptic space [68,69,72], where they might be ideally positioned to provide steady tonic inhibition [73]. Characterizing the remaining synaptic fraction, multi-labeling immunohistochemistry demonstrated the co-localization of punctate gephyrin and GlyR clusters—often detected using the pan-subunit antibody mAb4a—in close apposition to VGAT-positive presynaptic terminals [70,71]. This spatial configuration, with markers like Bassoon, identified functional synaptic sites, particularly on striatal large cholinergic (ChAT+) interneurons [70].

Sustained by the binding of the β subunit to gephyrin, which immobilizes the complex at the subsynaptic cytoskeleton [74], heteromeric αβ GlyR assemblies are expected to segregate predominantly into postsynaptic domains, though evidence suggests that this receptor–gephyrin complexing can occur prior to surface delivery [75]. Conversely, the major extrasynaptic fraction—particularly in the hippocampal CA1 area and cortex—lacks gephyrin co-localization and remains distant from VGAT-positive boutons [69,70]. To stabilize neural networks within the mature hippocampus, this predominant pool of extrasynaptic homomeric receptors coexists with a distinct population of β-subunit-containing heteromers [68] that either escape the synaptic cleft via lateral diffusion or are transiently trapped in extrasynaptic domains anchored by small, highly dynamic gephyrin microclusters [75].

The absence of gephyrin co-localization is generally regarded as a reliable structural indicator for α homomeric configurations. Early work by Levi et al. established that GlyR clustering is strictly dependent on gephyrin, whereby receptors remain diffuse and predominantly extrasynaptic in primary hippocampal neurons expressing α2 subunits without this scaffold [76]. Consistent with this principle, Bae et al. provided in vivo validation by showing that in Vmes neurons within the mesencephalic trigeminal nucleus, the expression of α3 subunits in the absence of gephyrin results in a somatic surface distribution suited for tonic modulation [77]. This organizational paradigm is challenged, however, by weak interactions between the α2 subunit and gephyrin, indicating a latent capacity for homomeric α2 complexes to aggregate within postsynaptic loci [78]. Supporting this possibility, native synaptic currents featuring homomeric kinetic hallmarks were recorded by Legendre et al. [79] from the hindbrain of 52 h-old zebrafish larvae. While this finding suggests that unanchored channel configurations might undergo functional synaptic activation, it must be interpreted cautiously; definitive in vivo evidence for completely homomeric synaptic transmission remains limited, as native kinetic signatures can be confounded by heterogeneous heteromeric assemblies.

The sparsity of GlyRs at forebrain synapses apparently explains the persistent difficulty in identifying them within purified synaptosomal fractions. Very recently, utilizing high-sensitivity SMLM (Single-Molecule Localization Microscopy) in an mEos4b-tagged GlyR knock-in mouse model, low-copy numbers of synaptic GlyRs were identified within hippocampal sub-regions, specifically the CA1, CA3, and dentate gyrus fields [80]. Dual-color SMLM revealed that 75% of inhibitory synapses completely lack GlyRs, whereas the remaining fraction integrates only 1 to 5 molecules within the postsynaptic gephyrin domain, suggesting that these sparse synaptic complexes might primarily serve to maintain structural integrity rather than mediating classical phasic transmission. In contrast, functionally relevant numbers of synaptic GlyRs were found at inhibitory synapses in the ventral striatum, highlighting that the balance between synaptic organization and the extrasynaptic pool is highly region-specific [80].

### 3.2. Pharmacological and Functional Profiling

Because anatomical distribution alone cannot fully differentiate coexisting extrasynaptic populations, the distinct pharmacological profile of these receptor pools serves as a definitive indicator of their homomeric or heteromeric assembly. While picrotoxin efficiently inhibits GABA_A_Rs, its effect on GlyRs depends strictly on their subunit composition. Synaptic αβ heteromers are picrotoxin-insensitive, a property attributed to the unique amino acid composition of the β subunit’s M2 transmembrane segment [36]. In contrast, homomeric α receptors lacking the β subunit remain highly sensitive to the blocker. Using single-channel recordings of α2 homomeric GlyRs, a pivotal study by Wang et al. demonstrated that picrotoxin reduces the mean open time of the channel, providing kinetic proof of an open-channel blockade [81]. Similarly, homomeric α3 configurations exhibit high susceptibility to picrotoxin inhibition. Work by Yang established the structural basis for this sensitivity, demonstrating that picrotoxin acts via pore-lining threonine residues shared across unanchored forebrain α2 and α3 homomers [82]. Zinc sensitivity further distinguishes the channel subtypes; while low nanomolar concentrations potentiate homomeric α currents, higher micromolar concentrations induce a potent inhibition [83].

Early functional evidence by Salling et al. utilized whole-cell patch-clamp recordings to identify a strychnine-sensitive tonic current in layer II/III pyramidal neurons [84]. Because the observed currents were abolished by picrotoxin, the study offered functional validation for the presence of homomeric GlyRs in this region. While the high expression of α2 initially suggested α2 assemblies, McCracken et al. clarified that the α3 subunit is specifically responsible for mediating the endogenous tonic currents observed within cortical layers [73]. Furthermore, utilizing the zinc chelator tricine, they demonstrated that the tonic conductance depends on ambient zinc for high-potency activation. These results align with findings by Jonsson et al. who showed by quantitative mRNA expression and immunohistochemical mapping in the rat nucleus accumbens and hippocampus that α2 and α3 remain the primary subunits in the forebrain, bypassing the α1-switch typically seen in the spinal cord [85].

Collectively, these data confirm that extrasynaptic homomeric GlyRs—specifically those composed of α2 and α3 subunits—are defined by their unique pharmacological sensitivity, gephyrin-independent localization, and specialized molecular variants as well as capacity for high-affinity activation, constituting a distinct and highly regulated inhibitory system in the forebrain and serving as a primary target for neuromodulation and pathological disruption.

## 4. Functional Aspects of Forebrain Extrasynaptic GlyRs

### 4.1. Tonic Inhibition

Extrasynaptic homomeric GlyRs contribute to a baseline, vesicular-release-independent tonic inhibitory conductance, serving to stabilize membrane potentials, reduce input resistance, shape firing thresholds, and regulate neuronal excitability to maintain network excitation–inhibition balance [1,34,73]. Such steady-state inhibitory tone arises from the persistent activation of extrasynaptic GlyRs by ambient levels of glycine, as well as by other endogenous agonists such as taurine. These ambient concentrations are maintained by a combination of synaptic spillover—where the agonist escapes the synaptic cleft during intense activity—and non-vesicular release from glial cells (Figure 1). Operating in the nanomolar to low micromolar range, this signaling relies primarily on high-affinity homomeric assemblies such as the edited α3 P185L variant that facilitates nanomolar ligand sensing [42] and the α2A homomer whose exceptionally slow kinetics sustain channel opening during prolonged exposure [45], with both mechanisms acting to maintain a constant inhibitory shunt. However, studies by Eichler et al. [16] and Winkelmann et al. [86] revealed that excessive tonic Cl^−^ conductance driven by these highly active edited configurations can alter the homeostatic electrochemical gradient, paradoxically leading to neurodegeneration and severe cognitive deficits, such as declarative memory impairment. Furthermore, Çaliskan et al. confirmed that this intracellular Cl^−^ overload directly impairs network oscillations required for spatial memory consolidation [87].

Physiologically, this tonic signaling provides high-precision gain control, suppressing spontaneous depolarization to dictate the threshold for voltage-gated calcium influx [34]. In the adult striatum, research by Molchanova et al. showed that tonically active α2-containing receptors paradoxically exert a depolarizing action in cells where intracellular Cl^−^ is elevated (*E_Cl_* above resting membrane potential), yet simultaneously provide a shunting conductance that reduces excitability regardless of the driving force direction [88]. This specific shunting mechanism dampens low-level excitatory noise, creating a localized voltage shield that safeguards neurons against chronic, excitotoxic Ca^2+^ overloads and subsequent mitochondrial dysfunction [89,90].

This buffering role was reinforced by McCracken et al., who identified matching tonic currents in the nucleus accumbens that stabilize membrane potentials against fluctuating excitatory inputs to set the neuronal rheobase [73]. Furthermore, in vivo microdialysis and calcium imaging revealed that ambient glycine levels—tightly regulated by Glycine Transporter 1 (GlyT1)—act as a constitutive inhibitory brake to prevent network hyperactivity [91,92]. Supporting this framework, Devoght et al., utilizing α2 knockout mouse models, demonstrated that the genetic absence of this subunit leads to disinhibited striatal signaling, confirming that these receptors normally modulate aberrant excitation [93].

Beyond structural protection, persistent inhibitory tone conserves metabolic energy by increasing the gap between the membrane potential and the firing threshold, preventing neurons from exhausting ATP on background noise. Because spikes are metabolically expensive, this silencing serves as a vital survival mechanism during cellular stress, preserving resources for critical processing rather than spontaneous activity [94]. Consequently, any disruption of tonic control can potentially lead to a collapse of the signal-to-noise ratio and glycine-buffering capacity, contributing to the neuronal hyperexcitability and Ca^2+^-mediated excitotoxicity that drive early-stage pathological network remodeling [95,96].

### 4.2. Crosstalk with Extrasynaptic NMDA Receptors

Beyond direct inhibition, extrasynaptic GlyRs engage in molecular crosstalk with NMDA receptors, where high-affinity homomeric assemblies, such as the edited α3 P185L variant, act as localized glycine scavengers. By sequestering ambient glycine, they restrict ligand availability at the co-agonist site, keeping extrasynaptic GluN2B-containing NMDA receptors silent during baseline activity [73,97]. Complementing this competition, GlyR-mediated tonic shunting exerts protective voltage control, reinforcing the voltage-dependent magnesium block of the NMDA receptor pore to safeguard neurons against accidental activation and subsequent Ca^2+^ influx [98]. Additionally, hippocampal GlyRs modulate glutamate release, providing a presynaptic homeostatic control mechanism that directly tunes excitatory neurotransmission. Research using hippocampal synaptosomes demonstrated that activating these presynaptic receptors modulates glutamate release, preventing excitatory circuits from reaching pathological saturation [99]. Expanding on this framework, Bohmbach et al. utilized computational modeling of CA1 pyramidal neurons to show that glycinergic tone enables non-linear synaptic integration by raising the dendritic spike threshold by a measured 8 to 12 mV, whereas its absence causes a collapsed dynamic range and a quantified 40 percent increase in over-excitation susceptibility [100]. This homeostatic defense is further supported by the work of Raiteri et al., which provided evidence in mouse models that mGlu1 receptor activation triggers endogenous glycine release to actively reinforce this presynaptic tone [101].

### 4.3. Modulation of Synaptic Plasticity and Glycine-Induced Long-Term Depression (Gly-LTD)

Extrasynaptic GlyRs contribute significantly to shaping long-term synaptic strength within the hippocampal CA1 region, extending their functional role into sustained synaptic remodeling. Within this circuit, elevated glycine levels—driven by astrocytic release or GlyT1 inhibition—trigger Gly-LTD, a non-canonical form of plasticity serving as a master homeostatic switch. Functional and structural analyses demonstrated that the activation of high-affinity extrasynaptic GlyRs drives the clathrin-dependent internalization of GluA1- and GluA2-containing AMPARs [102], a process facilitated by the dynamic flexibility and lateral diffusion of the gephyrin scaffold [65,67]. By coupling glycinergic tone to excitatory receptor density, this homeostatic mechanism effectively downscales synaptic transmission [102], protecting the circuit against excitotoxic damage and potentially maintaining a functional dynamic range during states of over-excitation.

### 4.4. Unconventional Excitatory Glycine Receptors

Expanding the classical inhibitory framework, unconventional GluN1/GluN3A excitatory glycine receptors (eGlyRs) operate within the mature brain as atypical members of the NMDA receptor family. While GluN3A is typically downregulated after development, accumulating investigations support that these receptors persist in specific adult circuits, representing a paradigm shift in the understanding of glycinergic transmission [103]. Gated solely by glycine and insensitive to magnesium block, eGlyRs translate ambient glycine into direct excitatory drive. In the ventral hippocampus, eGlyRs act as critical gatekeepers of emotional states. Optogenetic and electrophysiological evaluations established that eGlyR signaling in somatostatin-positive (SST) interneurons enhances theta-gamma (θ-γ) oscillations to suppress anxiety [104], while CRISPR-Cas9 knockdowns uncovered that eGlyR density serves as a molecular switch for stress adaptation [105]. Mechanistically, a dysfunctional eGlyR axis may be linked to the characteristic loss of SST interneurons [106] and the subsequent decoupling of θ-γ oscillations, thereby triggering network instability and anxiety as common early non-cognitive symptoms of AD—especially considering the reduced GluN3A expression measured in the brains of human patients [107]. Crucially, adult GluN1/GluN3A-containing receptors are diffusely expressed beyond these neocortical circuits. Work by Bossi et al. established that these channels participate in the consolidation of fear-associated memories within the basolateral amygdala [108]. Furthermore, they have been implicated in the development of aversive states, with a study by Otsu Y. et al. demonstrating that GluN1/GluN3A expression in the medial habenula is critical for generating negative emotional responses to unpleasant stimuli [109]. Together, these combined findings support a much broader physiological role for mature GluN3A signaling in adult emotional regulation and motivation [103].

### 4.5. Crosstalk with GABAergic Pathways

Extrasynaptic GlyRs stabilize overall network activity through a coordinated, reciprocal interface with the GABAergic system. To sustain this homeostatic framework, these receptors are strategically positioned on GABAergic axons and terminals, where they maintain a tonic Cl^−^ shunt [1] that prevents premature vesicle depletion and synaptic fatigue during sustained high-frequency activity. Such functional co-regulation depends on a shared transmembrane Cl^−^ gradient, driven by splitting the total inhibitory workload between both receptor types to prevent local intracellular Cl^−^ accumulation and subsequent depolarizing shifts [110]. Driven by the potassium–chloride cotransporter KCC2, this bioenergetic synergy actively counteracts circuit-wide hyperexcitability [111]. On a functional level, GlyR activation transiently reduces GABA_A_R-mediated currents [112], providing the dynamic inhibitory pressure required to generate the synchronized γ-oscillations essential for cognitive processing.

On a molecular level, this crosstalk is anchored by gephyrin, which determines the synaptic versus extrasynaptic distribution of both receptors [113]. While hexagonal gephyrin lattices ensure high-density GlyR clustering [114], recent single-molecule tracking and magnetic tweezers uncovered a direct molecular competition where GlyRs and GABA_A_Rs actively compete for the same anchoring sites [65]. Consequently, the failure of one inhibitory network may destabilize the other, potentially triggering network hyperexcitability that drives cognitive decline.

## 5. Implications in the Alzheimer’s Cascade

**Figure 2:**
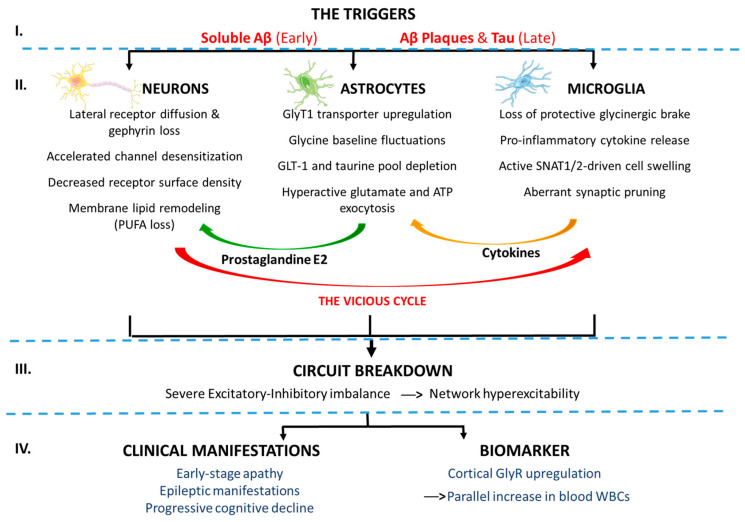
**Proposed mechanistic flowchart of glycinergic failure and network instability in Alzheimer’s disease.** The schematic illustrates the hypothetical progression of the glycinergic decline across four hierarchical levels: **Level I (Triggers):** Upstream pathology is presumed to initiate with soluble Aβ oligomers, progressing to late-stage Aβ plaques and pathological neurofibrillary Tau aggregates. **Level II (cellular dysregulation and a vicious cycle):** Pathological inputs are hypothesized to drive concurrent cellular defects. Neurons exhibit gephyrin scaffold loss, impaired microtubule transport, and lateral receptor diffusion, reducing GlyR surface density. Astrocytes undergo GLT-1/Taurine depletion and GlyT1 dysregulation, triggering Ca^2+^ hyperactivity and neurotoxic glutamate/ATP exocytosis. Microglia lose their GlyR homeostatic brake—inducing toxic cytokine release—while active SNAT1/2 transporters drive cell swelling and phagocytic synaptic pruning. Critically, a putative feed-forward loop is formed as microglial cytokines induce neurotoxic A1 astrocytes and activate a PGE_2_/PKA pathway, causing neuronal GlyR phosphorylation and surface receptor loss. **Level III (circuit breakdown):** These multicellular defects converge into network dysfunction, characterized by aberrant glial-mediated pruning and an E/I imbalance that promotes circuit hyperexcitability. **Level IV (translational readouts):** The network breakdown translates into clinical manifestations (early-stage apathy, progressive cognitive decline, and clinical epileptic manifestations) and a peripheral biomarker readout, where cortical GlyR upregulation correlates with circulating white blood cell (WBC) dynamics. Certain cell graphics in Level II are provided by Servier Medical Art, licensed under Creative Commons Attribution 4.0 International (CC BY 4.0).

AD is the leading cause of dementia worldwide [115], and is characterized by a complex spectrum of cognitive and non-cognitive alterations. While traditionally defined by amyloid-β (Aβ) and Tau accumulation, the clinical symptoms are increasingly understood as arising not from protein aggregation alone but from the downstream failure of synaptic homeostasis and cellular crosstalk. Central to this homeostatic collapse is a disruption in the excitatory/inhibitory (E/I) balance that frequently precedes significant neurodegeneration [116,117]. Clinical evidence strongly supports this model, as an estimated 10% to 22% of Alzheimer’s disease patients develop overt unprovoked seizures, whereas closer tracking with long-term electroencephalography reveals that up to 60% of patients exhibit silent, subclinical epileptiform activity over the course of the disease, reflecting a state of chronic neuronal hyperexcitability and impaired inhibitory control [118]. This network instability is subsequently embedded in a complex neuron–glia interplay, where chronic neuroinflammation and oxidative stress further exacerbate the underlying E/I imbalance [119]. While several aspects of this framework require further validation, Figure 2 presents a putative model linking hypothesized glycinergic deficits to progressive circuit dysfunction in AD.

### 5.1. Empirical Evidence for GlyR Alterations in Alzheimer’s Models

Although the role of the glycinergic system in the E/I balance and specifically in AD progression remains under-explored, emerging evidence suggests that the compromise of extrasynaptic GlyR signaling potentially contributes to a loss of circuit stability, representing a progressive failure that exacerbates network disruption and neuroinflammation across the entire AD continuum. This dysfunction appears to be driven by an initial biophysical impairment of receptor kinetics followed by a chronic structural depletion, especially of the extrasynaptic receptor pool. Electrophysiological recordings from acutely isolated rat hippocampal pyramidal neurons demonstrated that Aβ directly interferes with extrasynaptic GlyR kinetics through a multi-phasic inhibitory process. Utilizing the neurotoxic core fragment, Aβ (25–35), initial research established that picomolar concentrations of the peptide significantly accelerate the desensitization rate of glycine-induced currents [120]. Expanding these findings to the full-length Aβ (1–42) isoform, the same research team later confirmed that this kinetic modulation extends to other glycinergic agonists, including taurine and β-alanine [121]. These interactions were marked by a rapid acceleration of desensitization and a slowly developing suppression of peak current amplitude, particularly at the low ambient glycine concentrations characteristic of the extrasynaptic space. Collectively, Aβ-induced biophysical impairments likely led to a substantial reduction of the high-affinity tonic chloride shunt, increasing neuronal vulnerability to pathological depolarization.

Beyond this functional disability, chronic exposure to Aβ pathology triggers a progressive, structural depletion of the GlyR network. Validating this structural loss, biochemical and high-resolution immunofluorescence analyses in the APP/PS1 mouse model revealed that the protein depletion is progressive. While 12-month-old APP/PS1 mice exhibited a drastic reduction in α2 and α3 GlyR protein levels within the CA1 and dentate gyrus, 3-month-old mice showed no significant changes, suggesting that this structural decline follows the accumulation of Aβ-related pathology [69]. Furthermore, high-resolution imaging of double- and triple-labeled hippocampal sections indicated that the protein depletion primarily affected extrasynaptic receptors in the somatic layers of the hippocampus, a loss replicated in primary hippocampal neurons transfected with human APPswe [69].

In other in vivo AD models, GlyR alterations were observed already at early stages, frequently preceding the appearance of extracellular plaques, even positioning GlyR dysfunction as an upstream initiator of the disease cascade. Subcortical implications were documented in 6-month-old 2xTg AD mice, evidencing a selective 90% reduction in α2 GlyR mRNA within the nucleus accumbens [72]. Validated through GCaMP6 slice photometry, this receptor loss correlated with attenuated glycine-mediated calcium signals, impaired reward processing, and motivational decline. Specifically, behavioral assays demonstrated a reduced sucrose preference and decreased ethanol intake in the Drinking in the Dark (DID) paradigm, providing a biological basis for early-stage apathy. Beyond α2 and α3 isoforms, the causal link between receptor depletion and disease progression was substantiated for the α1 subunit in 6-month-old 5xFAD models. Ex vivo whole-cell current clamp recordings proved that functional impairment of extrasynaptic α1 subunits in the dentate gyrus drives pathological neuronal hyperactivity. By reducing the rheobase and lowering the current threshold for action potential generation, this loss of glycinergic tonic inhibition significantly increased granule cell firing frequency, thereby driving cognitive decline in spatial learning and memory tasks. Adeno-associated virus (AAV)-mediated ablation and point mutations (GlyR α1 S296A) further verified the role of these receptors. This specific serine-to-alanine substitution at position 296 disrupts a highly conserved residue critical for proper channel gating and allosteric signal transduction, rendering the channel functionally inactive. Implementing this mutation effectively prevented functional recovery and exacerbated hippocampal excitability [122].

While Aβ triggered widespread GlyR depletion in other circuits, the locus coeruleus (LC) appears to maintain a unique population of resilient receptors. Research uncovered that strychnine-sensitive GlyRs in the LC remain remarkably stable even when neighboring GABA_A_Rs are impaired [123]. This stability was observed both at the early stage of pathology in APP-PSEN1 mice (12–14 weeks) and in human post-mortem tissue from patients at advanced stages of the disease. Utilizing confocal microscopy and patch-clamp recordings, the study confirmed that GlyR expression stays intact despite chronic Aβ accumulation in this brain region. Of note, in the early-stage mouse model, pharmacologically activating these receptors with glycine successfully normalized neuronal hyperexcitability, highlighting the glycinergic system as a potential ‘rescue pathway’ during early disease progression.

Beyond these intrinsic channel failures, clinical autoimmune profiles further validate the functional degradation of glycinergic circuits. This pathophysiology was clinically reflected in human dementia patients, where autoantibodies directed against the GlyR co-occurred with profound axonal degeneration, thereby driving severe verbal memory recall deficits [124].

In conclusion, the spatiotemporal evidence indicates that the disruption of the extrasynaptic glycinergic axis within the AD cascade is neither uniform nor static. Instead, it manifests as a subunit-specific and region-dependent deterioration progressing from an initial biophysical impairment of receptor kinetics to a widespread structural collapse of the extrasynaptic inhibitory architecture, undermining both hippocampal cognitive circuits and subcortical reward pathways.

### 5.2. Putative Mechanisms Driving Glycinergic Collapse and Network Instability

Although direct molecular evidence in AD tissue is currently absent, the disruption of the extrasynaptic inhibitory framework can be mechanistically linked to further pathologies mainly through established structural and molecular dependencies. In this context, pathological tau aggregates might occupy a central role, leading to the impairment of both synaptic and extrasynaptic GlyRs via distinct biophysical and trafficking-dependent pathways. At the synapse, tau hyperphosphorylation drives cytoskeletal disintegration, potentially disrupting the sub-synaptic gephyrin scaffold [125,126]. This might be compounded by elevated CDK5 activity phosphorylating gephyrin at Ser270, which destabilizes its microtubule-binding affinity [62,63] and prompts proteolytic anchor degradation [127], causing heteromeric αβ GlyRs to laterally diffuse out of the cleft, impairing phasic inhibition. Concurrently, somatodendritic tau missorting might create microtubule transport impediments, blocking the kinesin-mediated exocytosis of homomeric α2- or α3-containing extrasynaptic vesicles [125], thereby reducing receptor surface density and compromising continuous glycinergic tonic inhibition.

The structural instability of extrasynaptic GlyRs in AD may be further compounded by a pathological remodeling of the membrane environment. Recent integrative data from The Neurolipid Atlas [128], obtained via high-resolution spatial lipidomics in tau pathology mouse models, demonstrated a localized loss of long-chain polyunsaturated fatty acid phospholipids and a concomitant accumulation of ceramides within Aβ-plaque-adjacent membrane regions, adding a critical biophysical dimension to this receptor vulnerability. While direct experimental verification is still required, these concurrent disruptions allow for the hypothesis that synergy between cytoskeletal transport failure and lipid-driven anchoring instability could alter the competitive balance between GlyRs and GABA_A_Rs for the remaining scaffold sites [65]. This proposed mechanism could ultimately promote a destabilizing redistribution of inhibitory receptors away from their proper functional domains.

Importantly, recent macroscopic neurophysiological assessments in human AD patients confirm a direct link between regional tau burden and local network instability. Magnetoencephalography modeling by Ranasinghe et al. demonstrated that increased excitatory time constants distinctly correlate with cortical tau deposition rather than Aβ, which drives a state of neural hyperexcitability [129]. While recent data by the same research team identified GABA_A_R deficits as a primary source of this hyperexcitability [23], the loss of the extrasynaptic glycinergic tonic brake emerges as a plausible, complementary mechanism that could critically contribute to network fragility.

### 5.3. Non-Neuronal Effects: Glycinergic Signaling in Glial Cells and Their Putative Roles in AD

The collapse of the glycinergic axis in AD likely extends to extrasynaptic GlyRs on glial cells, exacerbating the neuroinflammatory environment that drives neurodegeneration [119]. Within astrocytes and microglia, these receptors function as diffuse plasmalemmal sensors rather than synaptic mediators, monitoring interstitial glycine to modulate cellular phenotypes. Because many glial populations, unlike mature neurons, maintain high intracellular Cl^−^ concentrations via the NKCC1 transporter, GlyR activation induces membrane depolarization [130], transforming the channel into a molecular trigger for calcium-dependent signaling cascades [131].

Under physiological conditions, astrocytes act as primary metabolic partners regulating energy substrates and ionic homeostasis. Subunit distribution was shown to be regionally distinct; while spinal astrocytes express α1 subunits, forebrain populations predominantly utilize α2 and α3 assemblies [68]. Functionally, calcium imaging and patch-clamp recordings demonstrated that these receptors serve as a microtubule-dependent Ca^2+^ brake for the tripartite synapse [132]. By triggering a chloride-mediated shunt, glycine or taurine activation effectively suppressed ATP-induced Ca^2+^ transients, preventing uncontrolled excitatory gliotransmitter release; notably, pharmacological microtubule disruption with colchicine or nocodazole abolished this suppression, proving that proper membrane anchoring was essential for receptor functionality [132]. Furthermore, astroglial GlyRs fine-tuned the Ca^2+^ transients required for regulated D-serine exocytosis, acting as potent indirect modulators of NMDAR-mediated signaling [133].

In response to Aβ plaques, astrocytes undergo a transition into a neurotoxic reactive phenotype marked by GlyT1 dysregulation that drives pathological fluctuations in interstitial glycine levels [134,135]. This phenomenon eventually coincides with the profound reduction in the neuroprotective GlyR agonist taurine observed in the cerebrospinal fluid and brains of AD patients [136], thereby leaving extrasynaptic GlyRs chronically inactive and prone to structural collapse. Deprived of a functional glycinergic brake, and further compromised by the downregulation of glutamate transporter GLT-1 [137], AD astrocytes exhibit pathological, spontaneous Ca^2+^ hyperactivity that occurs independently of neuronal firing [138], inducing the aberrant vesicular release of neurotoxic glutamate and ATP, which in turn triggers local circuit instability [135]. Ultimately, this homeostatic failure might be compounded by a disruption in the astrocytic supply of glycine required for glutathione (GSH) synthesis, leaving neighboring neurons highly vulnerable to chronic oxidative stress [139].

The glycinergic system simultaneously extends to the innate immune response through microglial GlyRs—specifically α1, α2, α3, and β subunits—where their expression is directly coupled to the inflammatory state of the central nervous system [131]. Core experimental data using the BV-2 microglial cell line showed that the activation of these receptors by glycine or taurine functions as a molecular brake on pro-inflammatory M1 polarization, effectively attenuating the synthesis and release of neurotoxic cytokines such as TNF-α and IL-1β [140]. This suppression was largely mediated through the inhibition of NF-κB p65 and Hif-1α signaling pathways, effectively directing the microglial population toward a neuroprotective phenotype broadly characterized as M2-like, which favors tissue repair and homeostasis [140]. The critical nature of this glycinergic control was proved by subsequent studies showing that the pharmacological blockade of GlyRs with strychnine led to a spontaneous up-regulation of the TNFR1/NF-κB pathway, resulting in heightened microglial activation [141]. Beyond modulating cytokine output, GlyR activation enhanced microglial resilience during LPS-induced metabolic or inflammatory stress; specifically, glycine treatment improved cell viability and modulated apoptotic responses by stabilizing the GSH/GSSG ratio and reducing oxidative damage [142].

This protective cellular framework directly reflects the systemic immune response observed in vivo, where acute neuroinflammatory conditions induced a compensatory upregulation of GlyR α1 and α3 subunits within the cerebral cortex that closely paralleled the activation of Iba1-positive microglia [143]. Crucially, this landmark study characterized the ‘brain–glycinergic–blood’ axis by demonstrating that this central neuroinflammatory response corresponds precisely to a parallel increase in the same subunits within circulating white blood cells and peripheral macrophages. Utilizing flow cytometry and transcriptomic profiling, the authors established that peripheral leukocyte GlyR expression can serve as a reliable, minimally invasive circulating biomarker for the severity of central neuroinflammation and microglial activation in the forebrain [143].

In the context of AD, this glycinergic control system apparently undergoes a pathological breakdown. While Aβ directly triggers the release of pro-inflammatory cytokines such as IL-1β, TNF-α, and IL-6 from activated microglia [144], the concomitant failure of the glycinergic gating mechanisms—where glycine normally triggers hyperpolarizing chloride currents to dampen cellular reactivity—might further facilitate a sustained inflammatory state. This pro-inflammatory crosstalk is further exposed to extracellular, pathological tau aggregates, which act as potent drivers of tau-activated neuroinflammation via pattern recognition receptor signaling [145]. The resulting inflammatory loop promotes the activation of downstream NF-κB pathways, driving the conversion of healthy astrocytes into neurotoxic A1 phenotypes [146]. Specifically, Aβ triggers the upregulation of COX-2 and the subsequent hypersecretion of prostaglandin E2 (PGE2) to drive neuro-inflammatory cascade progression and downstream intracellular kinase activation [147]. Notably, PGE2-activated protein kinase A (PKA) was shown to directly phosphorylate the GlyR α3 subunit at Serine 346 in spinal neurons, reducing its Cl^−^ conductance and neuronal inhibitory capacity [12]. Concurrently, biochemical evidence in neuronal models has proven that these inflammatory signals trigger the physical removal of GlyRs from the membrane, where activation of PKA and PKC drives a significant 50 percent reduction in surface density through endocytosis [148].

Thus, in AD, microglial GlyR failure potentially contributes to a persistent E/I imbalance by perpetuating the neuroinflammatory cascades that culminate in glial-mediated synaptic pruning, a pathological progression established by recent high-resolution tissue studies [149]. Mechanistically, this glycinergic control of phagocytosis seems to operate via a distinct molecular dichotomy. First, the GlyR-dependent pathway regulates microglial phenotype and activation; microglial GlyR signaling suppresses neurotoxic M1 polarization [140]. Consequently, the loss of this GlyR-dependent signaling does not impair the physical engulfment machinery itself, but rather removes a critical homeostatic brake on microglial reactivity, thereby permitting unchecked, hyper-phagocytic phenotypes that drive aberrant synaptic pruning. Second, the acceleration of microglial particle engulfment by glycine is an entirely receptor-independent mechanism that is unaffected by strychnine [150]. Instead, this receptor-independent action is mediated by sodium-dependent neutral amino acid transporters SNAT1 and SNAT2, triggering Na^+^-coupled membrane depolarization and a cell volume expansion of approximately 9% necessary for pseudopodia formation [150,151]. Consequently, the loss of GlyR signaling may not impair the intrinsic phagocytic machinery itself, but rather removes the homeostatic brake on microglial reactivity, thereby permitting unchecked, hyper-phagocytic phenotypes that drive aberrant synaptic pruning.

## 6. Pharmacological Modulation of Glycinergic Transmission and Therapeutic Potential in AD

A feature of the glycinergic deficit in AD appears to be the spatial segregation between gephyrin-bound synaptic GlyR clusters and mobile extrasynaptic receptor populations. Rather than causing a uniform downregulation across the plasma membrane, Aβ conceivably targets these two domains through distinct mechanisms, leading to unequal rates of structural depletion whereby the unanchored extrasynaptic receptor pool exhibits a heightened vulnerability to neurotoxic insults compared to gephyrin-bound synaptic clusters [122]. To counteract the progressive loss of extrasynaptic GlyRs within AD circuits, the therapeutic rationale must center on a multifaceted pharmacological framework targeting receptor expression and trafficking, allosteric modulation of channel gating, and optimization of the ambient glycinergic tone to ultimately re-establish homeostatic network inhibition.

Unfortunately, pharmacological interventions specifically targeting inhibitory GlyR channels in AD remain remarkably sparse, with current literature restricted almost exclusively to a narrow spectrum of plant-derived molecules and their synthetic derivatives. Historically, investigations into glycinergic modulation in dementia were directed toward the glycine co-agonist binding site of excitatory NMDA receptors using compounds like milacemide, a framework functionally distinct from the regulation of inhibitory Cys-loop channels. This historical strategy aimed to enhance cognition by potentiating glutamate-mediated calcium influx; however, these trials failed primarily due to a lack of efficacy [152], while highlighting concerns that overactivating this excitatory co-agonist pathway carries a mechanistic risk of accelerating excitotoxic neuronal death. Ultimately, these clinical outcomes underscore the necessity of separating the excitatory modulation of ionotropic glutamate complexes from the activation of classical, inhibitory Cys-loop GlyRs that protect vulnerable circuits by driving membrane hyperpolarization.

Addressing structural preservation and trafficking pathways via an indirect modulatory approach, low-dose treatment with the antimalarial drug artesunate was shown to counteract receptor degradation in the APP/PS1 mouse model in a strictly subunit-specific manner [69]. This intervention effectively restored the severe loss of the extrasynaptic α3 GlyR pool to wild-type levels within the hippocampus of 12-month-old mice, while leaving α2 subunit levels unaffected [69]. This selectivity may reflect a dose-dependent hormetic action; while high concentrations of artemisinins were shown to disrupt scaffolding, low-dose artesunate likely stabilizes the wider inhibitory framework, including gephyrin and GABA_A_Rs [153,154]. Because artemisinins occupy the receptor-binding pocket of gephyrin [114], this selective rescue potentially involves an indirect upstream pathway that effectively restores depleted synaptic proteins and reduces toxic APP processing, as already evidenced in this mouse model [154]. Relieved from Aβ-induced stress, the neurons might safely utilize their trafficking machinery to repopulate the extrasynaptic domain with newly synthesized homomeric α3 subunits while preventing their pathological internalization and endocytic clearance. Crucially, the well-documented capacity of artemisinins to suppress microglial overactivation and downregulate pro-inflammatory cytokines under chronic Aβ exposure may play a complementary, protective role in this compartment-specific modulation [155], by dampening overall membrane stress, although probably remaining restricted to α3 due to distinct clear-and-rescue pathways.

Complementing these structural rescues through direct pharmacological actions on the channel complex, chronic administration of cannabidiol and its synthetic, non-psychoactive derivatives was recently reported to significantly mitigate cognitive deficiency and hippocampal amyloid-beta pathology by potentiating the function of inhibitory extrasynaptic GlyRs within the dentate gyrus of 6-month-old 5xFAD mice [122]. This interaction enhanced receptor sensitivity to low ambient glycine levels, driving a robust Cl^−^ influx that stabilized the glycinergic shunt and conceivably suppressed spontaneous Ca^2+^ hyperactivity. Importantly, this potentiation rescued GlyR signaling from prostaglandin E2-mediated inflammatory suppression, potentially preventing downstream complement-mediated synaptic pruning [122]. To establish causality in vivo, the authors used adeno-associated virus vectors to ablate dentate gyrus α1 subunits or introduce an S296A mutation within the TM3 helix. Both interventions completely abolished cannabidiol’s benefits, proving that recovery depended on functional α1 signaling and rendering the allosteric potentiation strictly dependent on membrane cholesterol accessibility within lipid rafts. Crucially, the validation of the α1 S296 binding pocket demonstrated that these neuroprotective, tonic inhibitory currents could be successfully reinforced independently of classical cannabinoid receptors [122]. Consequently, this study pinpointed the α1 GlyR-cannabidiol interface as a versatile target to normalize neurodegenerative alterations in neurotransmitter signaling, metabolism, and neuroinflammation.

Beyond cannabinoids and artemisinin derivatives, other chemical classes demonstrated direct, allosteric modulatory efficacy at ionotropic GlyRs within the CNS in preclinical studies. For instance, endogenous neurosteroids such as pregnenolone sulfate and the antiparasitic macrocyclic lactone ivermectin emerged as highly potent positive allosteric modulators capable of directly amplifying tonic inhibitory currents [156]. In a recent study using a streptozotocin-induced rat model, chronic intraperitoneal administration of ivermectin attenuated cognitive decline in behavioral memory tests, with histological evaluation confirming that this treatment significantly reduced Aβ-plaque accumulation and reduced markers of neuronal apoptosis [157]. Although this in vivo study established the overall neuroprotective outcome, independent biophysical and high-resolution structural analyses confirmed that the underlying molecular mechanism of ivermectin relies on its binding to allosteric pockets within the transmembrane domain of strychnine-sensitive GlyRs, effectively stabilizing the open pore conformation, slowing accelerated desensitization, and augmenting the whole-cell Cl^−^ response under neurotoxic conditions [156,158]. However, ivermectin lacks target specificity and simultaneously modulates GABA_A_ receptors, purinergic P2X4 and P2X7 receptors, and G-protein-gated inwardly rectifying potassium channels. Because these overlapping target pathways can independently regulate neuroinflammation and cellular survival, the multi-system neuroprotective outcomes observed in vivo cannot be definitively attributed to glycinergic signaling alone [159].

Future clinical trials must evaluate whether artemisinin derivatives, phytocannabinoids, and ivermectin can replicate these preclinical neuroprotective mechanisms in human cohorts. Crucially, all three classes offer a major translational advantage as well-characterized, clinically approved compounds for other indications—artemisinins as antimalarials [160], specific cannabinoids for severe epilepsies [161], and ivermectin as an antiparasitic drug [162]. This established human safety and pharmacokinetic profile should bypass early-phase regulatory hurdles, enabling rapid drug repositioning for AD. However, a critical pharmacokinetic contrast remains for human translation, as artemisinin derivatives and phytocannabinoids inherently cross the blood–brain barrier (BBB) with high efficiency, whereas classical macrocyclic lactones encounter a P-glycoprotein-mediated efflux barrier at the BBB [163]. Consequently, translating ivermectin’s neuroprotective animal doses to humans will require optimized delivery systems or pump modulators to achieve central therapeutic thresholds safely without peripheral toxicity. Of further relevance, because these drug classes exploit entirely different biophysical pathways—ranging from upstream gephyrin-scaffolding stabilization to direct allosteric modulation of specific α subunit conformations—their simultaneous clinical availability establishes a strong rationale for multi-target combination therapies, offering a synergistic strategy to arrest early neurodegenerative progression.

Beyond these specific classes, numerous studies describe several other allosteric modulators that serve as valuable templates for correcting network dysfunction. Comprehensive reviews and milestone updates on complex receptor-associated protein networks summarize how these diverse structures may influence transmission dynamics [34,164]. For instance, certain tropeines can act as highly potent potentiators of α homomers, while broad-spectrum general anesthetics like propofol appear to enhance channel open probability through distinct inter-subunit pockets within the transmembrane domain. Furthermore, recent drug discovery efforts suggest that synthetic tricyclic sulfonamides, such as the prototypical modulator AM-1488, can operate as effective, non-selective positive allosteric modulators that successfully stabilize channel gating [165]. Together with novel positive modulatory RNA aptamers developed by Shalaly et al. [166], these diverse molecular tools highlight that reinforcing glycinergic inhibition remains a promising avenue to combat the electrical instability driving progressive neurodegeneration.

Alternatively, targeting endogenous co-agonists like taurine or β-alanine remains a viable secondary axis to reinforce remaining functional extrasynaptic receptor pools against Aβ insults. Oral taurine supplementation was recently shown to suppress Aβ accumulation and mitigate hippocampal neuroinflammation in 5xFAD mice [167]. Mechanistically, these neuroprotective actions are likely mediated through strychnine-sensitive extrasynaptic GlyRs, where both taurine and β-alanine act as functional agonists to restore homeostatic tonic currents [168]. However, patch-clamp analyses demonstrated that even picomolar concentrations of Aβ directly accelerate the desensitization kinetics of these receptor currents when activated by taurine or β-alanine, limiting monotherapeutic efficacy [121]. Therefore, while manipulating endogenous β-amino acid pools counteracts neuronal hyperexcitability, long-term translational success will require adjunctive interventions capable of preventing Aβ-mediated GlyR desensitization.

To complement direct modulation, sustaining an ambient extrasynaptic transmitter tone via targeted release or reuptake interventions represents a parallel strategy to recruit non-canonical neuroprotective pathways. Specifically, activating presynaptic metabotropic glutamate receptor one (mGluR1) directly upregulates hippocampal glycine release, a homeostatic mechanism fully abolished in mGluR1-deficient mice [101], establishing presynaptic mGluR1 as another candidate to harness endogenous glycinergic protection against Aβ neurotoxicity. In parallel, inhibiting GlyT1 via intrahippocampal pretreatment with the sarcosine derivative NFPS (N-[3-(4′-fluorophenyl)-3-(4′-phenylphenoxy)propyl]sarcosine) was recently shown to prevent transmitter reuptake, thereby mitigating cognitive deficits, neuroinflammation, and subsequent neuronal damage in Aβ-induced AD mouse models [169]. Despite these promising preclinical strategies, this therapeutic framework faced a severe translational setback when the selective GlyT1 inhibitor iclepertin failed as a monotherapy to achieve cognitive improvements in patients with Alzheimer’s disease [170], confirming that single-target approaches cannot overcome the systemic complexity of AD, shifting the future therapeutic focus toward multi-target combination strategies.

## 7. Conclusions and Future Perspectives

The contemporary evidence synthesized in this review supports the concept of subunit-selective GlyR modulation as a viable disease-modifying approach to counteract the E/I imbalance driving AD pathology. Beyond traditional amyloid and tau interventions, stabilizing GlyR-mediated inhibitory tone offers a dual mechanistic benefit: it directly mitigates excitotoxic neuronal damage and indirectly modulates the NMDA receptor co-agonist site by regulating ambient glycine availability to rescue synaptic plasticity.

Translating these preclinical insights into successful clinical strategies requires overcoming key structural, pharmacokinetic, and clinical milestones. First, drug discovery must exploit detailed structural conformations of hippocampal receptor sites to engineer highly potent synthetic positive allosteric modulators with optimized receptor affinity and central nervous system specificity. Parallel to this, pharmacokinetic refinement must focus on developing novel small molecules or indirect transport modulators to overcome BBB limitations and avoid peripheral motor side effects.

Ultimately, establishing dedicated human clinical trials designed specifically to monitor GlyR target engagement and cognitive outcomes is mandatory to bridge the translational gap. Direct clinical validation for this specific mechanism remains absent, as current human data on pleiotropic natural compounds like cannabinoids are heavily constrained. Existing studies are limited strictly to managing non-cognitive neuropsychiatric agitation via synthetic agents like dronabinol [171] or providing generic, broad neurosedative effects through low-dose THC formulations [172]. Furthermore, artemisinins lack clinical testing in dementia entirely. However, it remains imperative to underscore that the widespread clinical use of these compounds in populations with epilepsy, spasticity, or chronic parasitic infections could offer a unique theoretical framework for future retrospective cohort studies to evaluate whether long-term GlyR modulation correlates with any unintended, secondary neuroprotective benefits or altered dementia risk profiles in these patient populations. Only overcoming these hurdles will clarify whether GlyR modulation can transition from a promising preclinical concept to a validated clinical strategy for Alzheimer’s pathology.

## Data Availability

No new data were created or analyzed in this study. Data sharing is not applicable to this article.
